# Structural and Functional Connectivity Substrates of Cognitive Impairment in Multiple Sclerosis

**DOI:** 10.3389/fneur.2021.671894

**Published:** 2021-07-08

**Authors:** Jian Zhang, Rosa Cortese, Nicola De Stefano, Antonio Giorgio

**Affiliations:** Department of Medicine, Surgery and Neuroscience, University of Siena, Siena, Italy

**Keywords:** multiple sclerosis, structural connectivity, functional connectivity, cognitive impairment, substrates

## Abstract

Cognitive impairment (CI) occurs in 43 to 70% of multiple sclerosis (MS) patients at both early and later disease stages. Cognitive domains typically involved in MS include attention, information processing speed, memory, and executive control. The growing use of advanced magnetic resonance imaging (MRI) techniques is furthering our understanding on the altered structural connectivity (SC) and functional connectivity (FC) substrates of CI in MS. Regarding SC, different diffusion tensor imaging (DTI) measures (e.g., fractional anisotropy, diffusivities) along tractography-derived white matter (WM) tracts showed relevance toward CI. Novel diffusion MRI techniques, including diffusion kurtosis imaging, diffusion spectrum imaging, high angular resolution diffusion imaging, and neurite orientation dispersion and density imaging, showed more pathological specificity compared to the traditional DTI but require longer scan time and mathematical complexities for their interpretation. As for FC, task-based functional MRI (fMRI) has been traditionally used in MS to brain mapping the neural activity during various cognitive tasks. Analysis methods of resting fMRI (seed-based, independent component analysis, graph analysis) have been applied to uncover the functional substrates of CI in MS by revealing adaptive or maladaptive mechanisms of functional reorganization. The relevance for CI in MS of SC–FC relationships, reflecting common pathogenic mechanisms in WM and gray matter, has been recently explored by novel MRI analysis methods. This review summarizes recent advances on MRI techniques of SC and FC and their potential to provide a deeper understanding of the pathological substrates of CI in MS.

## Introduction

It has been nearly 150 years since Charcot described cognitive impairment (CI) in multiple sclerosis (MS) patients as “enfeeblement of memory” and “concepts formed slowly” (1). The importance of CI in MS was reinforced a few decades ago, after a long period of underestimation (2). CI in MS patients can affect multiple domains including attention, information processing speed (IPS), memory, and executive control (3, 4) and may be present since the early disease stages, being more prevalent in the progressive forms (5) (see Box 1 for a definition of MS phenotypes). Recently, in order to overcome the heterogeneity of CI in MS, some studies have proposed cognitive phenotypes, characterized by the prevalent impairment of a specific cognitive domain, based on predefined cutoff values (6, 7) or latent profile analysis (8). Furthermore, the involvement of cognitive reserve has been suggested to partly explain the “clinicoradiological paradox” in MS patients without CI despite the evidence of brain damage (9–13).

Box 1 **MS phenotypes**

**Clinically isolated syndrome (CIS)**

A monophasic clinical episode with patient-reported symptoms and objective findings reflecting a focal or multifocal inflammatory demyelinating event in the central nervous system, developing acutely or subacutely, with a duration of at least 24 h, with or without recovery, and in the absence of fever or infection, similar to a typical MS relapse (attack and exacerbation) but in a patient not known to have MS (14).

**Relapsing–remitting MS (RRMS)**

Presence of relapses with stable neurological disability in between them (14).

**Secondary progressive MS (SPMS)**

Progressive course following an initial relapsing–remitting course (14).

**Primary progressive MS (PPMS)**

Progressive course from disease onset (14).

Magnetic resonance imaging (MRI) may contribute to improve the current partial understanding of the pathogenic mechanisms of CI in MS. Over the last decade, several MRI measures have been proposed as biomarkers of CI in MS, including white matter (WM) lesion load and distribution, gray matter (GM) lesions, and cortical and deep GM atrophy (9, 15).

However, abnormalities in MS are not simply confined to a single brain region but rather tend to spread via axonal pathways, thus involving other regions (16). More recently, taking into account the complex topological organization of the human brain, advanced MRI techniques assessing structural connectivity (SC) or functional connectivity (FC) have been developed and applied to various neurological conditions, including MS (17).

The aim of this review was to summarize the recent applications in MS of MRI-based SC and FC approaches to the assessment of the pathogenic substrates of CI in different cognitive domains, starting with a brief methodological description. Finally, future directions and challenges will be discussed.

For all these purposes, this review included scientific literature of the last 10 years from PubMed using the search terms “cognition,” “cognitive impairment,” “cognitive deficits,” “cognitive decline,” “cognitive dysfunction,” “multiple sclerosis,” “neuropsychological evaluation,” “connectivity,” “functional connectivity,” “structural connectivity,” “network,” “cognitive phenotypes,” “cognitive reserve,” “fMRI,” “resting-state fMRI,” “diffusion MRI,” “diffusion tensor imaging,” “tractography.”

## Assessment of Brain Connectivity

### Measuring SC

**Figure 1:**
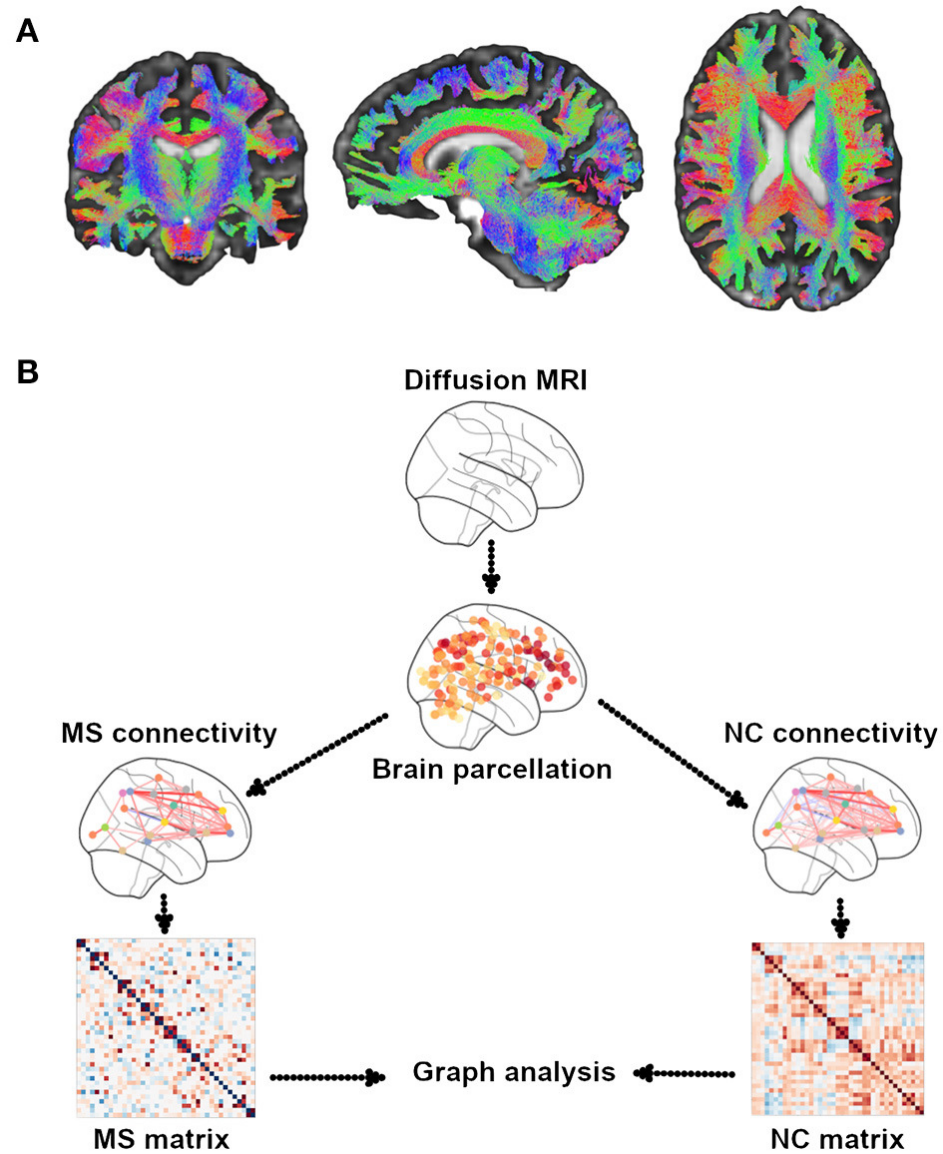
Illustrative example of WM tractography. **(A)** Different colors show the three systems of WM tracts: red for commissural (laterolateral direction), green for association (anterior–posterior direction), and blue for projection (superior-to-inferior direction). WM tractography were overlaid onto MNI standard brain. **(B)** A general overview of the pipeline of graph theory analysis for the assessment of structural brain networks. A network or a graph is a collection of vertices (nodes) and corresponding pairwise connections (edges). A comprehensive set of all pairwise connections in the brain defines the topology of a brain network, providing a complete connectivity diagram of all connections among nodes and edges, that is, a connectome. There are four essential steps in performing a graph theory analysis: (1) defining nodes: nodes are brain regions of interest (ROIs), typically derived from an anatomical parcellation of an imaging dataset; (2) defining edges: edges reflect the relationship between each node pair; they can be streamline connections derived from DTI tractography; (3) constructing a network: this step integrates all the information from nodes and edges in order to generate a complete connectivity map; the simplest representation of a network is using a two-dimensional matrix (i.e., a connectivity matrix); (4) graph theory analysis: currently, the most commonly used method to assess the characteristics of a network; it provides various measures of network topology. MS, multiple sclerosis; NC, normal controls.

Diffusion MRI is a type of sequence that is sensitive to the random microscopic motion of water molecules (18), thus providing information on the microstructure of WM fiber tracts noninvasively. Since the introduction of diffusion tensor imaging (DTI) (19), which assumes a Gaussian diffusion of water molecules, images, and corresponding indices derived from the tensor model, such as fractional anisotropy (FA) and mean, axial, and radial diffusivities (20), were used to assess structural integrity along tractography-derived WM fiber tracts, a proxy for SC (18, 21, 22) (Figure 1). Because of the limitations of traditional DTI regarding regions with crossing fibers and multiple fiber orientations within a single voxel, alternative diffusion methods have been proposed. They include diffusion kurtosis imaging (DKI) (23), diffusion spectrum imaging (DSI) (24), high angular resolution diffusion imaging (HARDI) (25), and neurite orientation dispersion and density imaging (NODDI) (26), which assess, respectively, the non-Gaussian behavior of water diffusion, the likelihood of water diffusion along any space direction, the orientation density function using less sampling intensive spherical q-space acquisitions, and the angular variation of neurite orientation. These methods offer the potential added value of a higher sensitivity to pathological changes over traditional DTI (27, 28). However, long scan time and mathematical complexities have thus far hindered their use in the clinical setting.

Two main approaches of tractography exist, referred to as deterministic and probabilistic (21). The former reconstructs WM fibers assuming a single orientation within each voxel, whereas the latter assumes an orientation distribution of such fibers (21). SC across the brain is typically built up by first defining a pair of parcellated regions (see Box 2 for the parcellation details) and then running tractography and finally assessing connectivity measures from the connecting WM streamlines (21). Each region is defined as a “node,” whereas WM connections are considered as “edges” of the structural network (27, 28). Within this framework, graph analysis can be performed on the SC matrix and allows deriving various network measures of integration (path length, global efficiency), segregation (clustering coefficient, transitivity, local efficiency, modularity), centrality, motifs, resilience (degree, assortativity coefficient), and other features (small worldness, rich club coefficient) (28, 29) (see Box 3 for details on graph theory measures). These measures help unveil the topological features of brain structural networks and can be used to study the relationship with cognitive functions (30). In contrast to graph analysis, data-driven mapping approaches such as independent component analysis (ICA), a multivariate method identifying single brain structural networks (31, 32), and nonnegative matrix factorization (NMF), an unsupervised technique based on structural network parcellations from DTI data (33), may be used, by providing a different way of assessing disrupted SC in pathological conditions (31, 32) (see Box 2 for strengths and limitations of SC assessment).

Box 2 **Summary of different approaches assessing brain connectivity**

**Structural connectivity**

***Graph theory methods***

The connectivity matrix, a squared N × N matrix representing connectivity between nodes, is typically constructed from a combination of brain tractography and any type of parcellation (21):

*Anatomical parcellation*: Node definitions based on *a priori* anatomical information, such as sulci and gyri, or anatomically predefined ROI (34)
*Strength:* Rapid and intuitive parcellation
*Limitations:* Low resolution, large variations in node size

*Random parcellation*: Brain is randomly parcellated into discrete nodes of similar size (34)
*Strength:* Minimizes node size variations
*Limitations:* Unclear validity/reliability

*Functional parcellation*: Node definitions based on *a priori* functional information, such as coordinates of peak activations or meta-analytic results (34)
*Strength:* Hypothesis-driven, equal node size
*Limitations:* Definitions are data-specific, may miss some regions, difficult to apply to diffusion MRI data

*Voxel-based parcellation*: Each image voxel represents a distinct node (34)
*Strength:* Data-driven, high resolution
*Limitations:* Computationally intensive

***Data-driven methods***

Model-free; connectivity is identified by the multivariate methods:

*Independent component analysis*: Performs a linear decomposition on the whole brain tractography matrix for identifying structural connectivity (32)
*Strength:* Data-driven
*Limitations:* The estimated independent components and the respective mixing matrix can contain both positive and negative values, leading to challenges in the interpretation of negative weights.

*Nonnegative matrix factorization:* An unsupervised technique for extracting connectivity components from diffusion MRI data, both at the group and individual level (33)
*Strength:* Data-driven, easy for interpretation
*Limitations:* Biased decomposition, computationally intensive

**Functional connectivity**

Statistical dependency (i.e., Pearson correlation coefficient) between signals measured from different “brain units” is thought to be indicative of FC (35), based on:

- Task fMRI (36)
  *Strength:* Directly reveals differences related to a task (e.g., cognitive, motor)
  *Limitations:* Patients may have difficulty in completing the scan, interpretation of fMRI results during cognitive tasks can be difficult when task performance differs across patients
- Resting fMRI (35)
  *Strength:* Easier for patients to complete the scan
  *Limitations:* It may provide just a partial picture of the brain's functional architecture, missing the functional reorganization shown by task fMRI

*Static edge-based functional connectivity:* Edge-based summary measures include full or partial correlation and mutual information (35)
*Strength:* Easy to implement
*Limitations:* Cannot provide direction information of FC, interpretation challenges in case of brain pathology

*Effective connectivity*: Evaluates the directionality and strength of FC between pairs of “brain units” (35)
*Strength:* It can provide direction information of connectivity
*Limitations:* Difficult to find an appropriate model for fast changes in effective connectivity

*Dynamic functional connectivity*: Reflects variations in FC over time (35)
*Strength:* Captures time-varying FC
*Limitations:* Signal-to-noise ratio of MRI data may be a practical limitation for FC assessment

Box 3 **Graph analysis glossary in the review**

**Node**

Neurons and/or brain regions (37)

**Edge**

Functional (29) or structural (38) relationships between brain regions

**Nodal strength**

Sum of the weighs across all connections associated with that node (39)

**Path length and efficiency**

*Path length* is the minimum number of edges that must be traversed to go from one node to another (28).

The average inverse shortest path length is a related measure known as the *global efficiency* (29).

Path length and global efficiency measure the ability of parallel information exchange across the whole network (40).

The *local efficiency* of a particular node is the inverse of the average shortest path connecting all neighbors of that node, measuring the information transfer in the immediate neighborhood of each node (41).

**Clustering coefficient and transitivity**

The fraction of triangles around an individual node is known as the *clustering coefficient*, and is equivalent to the fraction of the node's neighbors that are also neighbors of each other.

Clustering coefficient reflects the network segregation (29), the ability for specialized processing to occur within interconnected groups of brain regions (41).

The *transitivity* is the ratio of triangles to triplets in the network and is an alternative to the clustering coefficient (29).

**Modularity**

It measures the quality of division of a network into modules (41).

**Centrality**

It measures the relative importance of a node or edge within the overall architecture of a network (37).

**Motif**

Small (e.g., three or four nodes) patterns of local connectivity that occur in the network with a statistically surprising frequency (29)

**Degree**

Number of edges attached to a given node (37)

**Hub**

A node occupying a central position in the overall organization of a network (37)

**Rich club**

A set of high-degree nodes in a network to be more densely interconnected than expected on the basis of their node degree alone (37). The *rich club* effect of brain networks plays an important role in the information transmission across the brain (41, 42)

**Feeder**

Connections linking rich club nodes to nonrich club nodes (43).

**Assortativity and hierarchy**

*Assortativity* is a measure of the tendency for nodes to be connected to other nodes of the same or similar degree (28).

*Hierarchy* is the tendency of hubs to connect to nodes that are not otherwise connected to each other (44).

Increased assortativity and reduced hierarchy indicate an impaired wiring efficiency at a system level (44).

**Mean network degree**

The average degree of all network nodes and a measure of network density (29)

**Module efficiency**

Evaluating the communication efficiency both within and between structural networks (45). Intramodule efficiency: measures the global efficiency of the parallel information transfer within the module; intermodule efficiency: measures the global efficiency of the parallel information transfer between two different modules (45). Module: a group of nodes that maintains a large number of mutual connections and a small number of connections to nodes outside their group (37)

**Small worldness**

A network that shows a level of clustering higher than that observed in random networks and an average shortest path length that is equal to that observed in random networks (37)

**Network efficiency**

Assessment of the exchanging information performance of small-world brain functional networks (40)

**Communicability**

Measure of network integration. It accounts for the contribution of all possible walks between a pair of nodes, reflecting a network's capacity for parallel information transfer under a diffusion model of information flow (46). Walk: a path in a network that is allowed to visit the same nodes and edges on multiple occasions (46)

### Measuring FC

Functional MRI (fMRI) is a well-established method able to detect at the level of GM regions changes in the blood oxygenation level–dependent (BOLD) signal, which indirectly reflects neuronal activation *in vivo* (47). Two fMRI paradigms exist: the first is task based, assessing the brain regions activated during a specific task (e.g., cognitive, motor) (48), whereas the second is resting-state fMRI, measuring the similar spontaneous fluctuations of the BOLD signal between brain regions—FC—reflecting “intrinsic” functional relationships (35).

**Figure 2:**
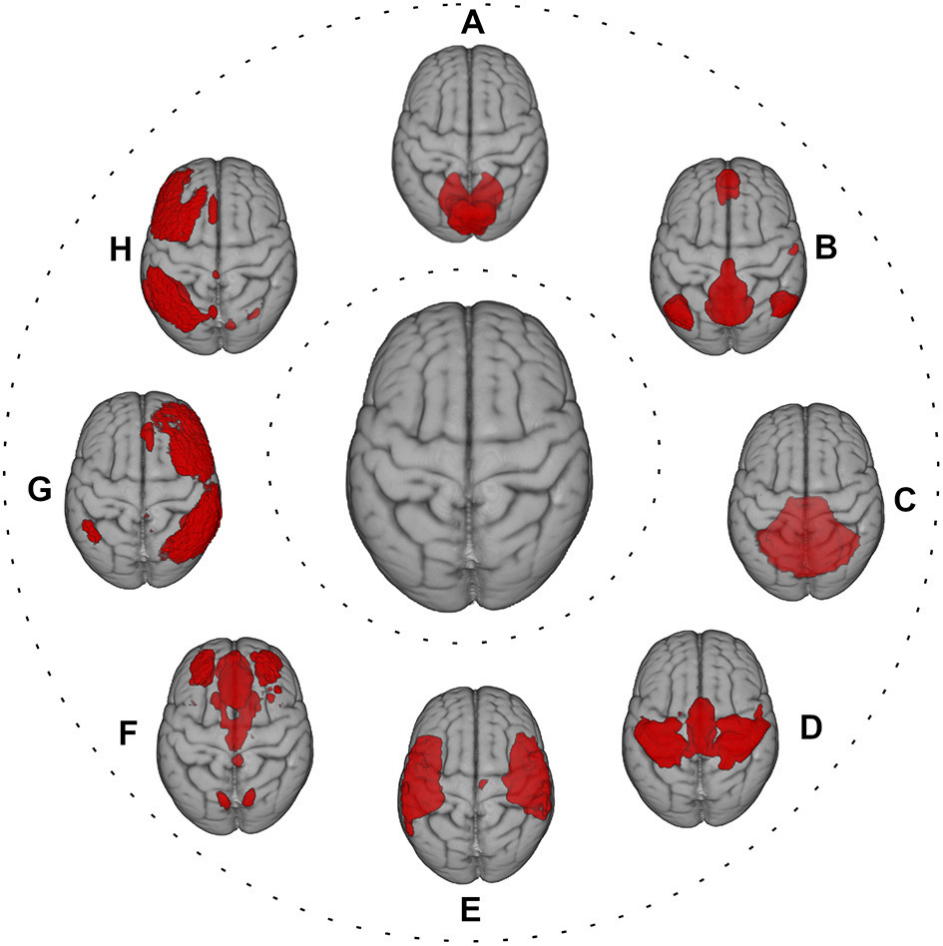
Red color shows the most representative resting-state networks, reflecting large-scale functional patterns, overlaid onto MNI standard brain. **(A)** visual network; **(B)** default mode network; **(C)** cerebellum network; **(D)** sensorimotor network; **(E)** auditory network; **(F)** executive control network; **(G)** right frontoparietal network; **(H)** left frontoparietal network.

A “brain unit” can be viewed as a spatially defined functional processing unit at different levels, including parcellated brain regions, regions of interest (ROIs), or resting-state networks (Figure 2) (35, 49, 50). In this context, FC can be considered in terms of statistical similarity (i.e., full or partial correlation, mutual information) between signals measured from pairs of brain units (35). For instance, after defining an ROI as “seed,” a correlation map with another ROI or the whole brain can be estimated (35). Moreover, FC can be assessed, similarly to SC, in the graph theory framework, using the measures listed in the previous paragraph. Finally, FC may be derived at voxel level, using dual regression on the ICA decomposition maps of resting fMRI (51).

Context-dependent connectivity between brain units during a task fMRI (49) and intrinsic connectivity between time series of brain units during resting fMRI (35) can also be obtained. In addition to the traditional “static” connectivity, variations in the FC over time—dynamic FC—and in the directionality and strength of FC between pairs of brain units—effective connectivity—can be assessed (35, 52) (see Box 2 for the strengths and limitations of FC assessment).

In order to investigate the substrate of CI in MS, FC may be used directly in the statistical models or fed into the graph theory framework to extract corresponding measures (30, 53).

## Connectivity Substrates of CI in MS

### Global Cognition

Various tools are available to explore cognition in MS (4, 54), from short screening tests to full neuropsychological batteries covering a wide range of cognitive performances (54–56). The former includes the Brief International Cognitive Assessment for Multiple Sclerosis (57) and the Multiple Sclerosis Outcomes Assessment Consortium (58), whereas the latter comprises the Brief Repeatable Battery of Neuropsychological tests (59) and the Minimal Assessment of Cognitive Function in MS (56). Global CI in MS can be defined in different ways: (i) performance ≤1.5 to 2 standard deviations (SDs) from the mean normative values in 20 to 30% of tests, (ii) impairment ≥1.5 to 2 SDs in at least two cognitive domains, (iii) use of composite scores, (iv) a combination of the above systems (60).

#### Structural Connectivity

Relapsing–remitting (RR) and secondary progressive (SP) MS patients with CI both showed a widespread reduction in two key measures of SC, such as local efficiency and nodal strength, suggesting the presence of a network collapse or its inability to compensate for such impairment (61). It is thought that CI in MS may be the result of a “disconnection syndrome” (17, 62). Such hypothesis was investigated in RRMS patients at whole-brain level in terms of path length, and it was found that impaired long-range rather than short-range FA-based connections had stronger correlation with decreased structural network efficiency, as well as with worse global CI measured by a composite score. These findings suggest that MS pathology mainly interrupts structural pathways connecting remote brain regions playing an important role for global cognition (63).

#### Functional Connectivity

Disruption of global FC, as shown by both reduction in mean network degree, global efficiency and hierarchy, and increase in path length and assortativity, contributed to distinguish MS patients with CI [benign MS (BMS), RR, SP] from those without CI and healthy controls (64).

In pediatric RRMS, patients with preserved cognition showed, compared to healthy controls and patients with CI, an increased FC in the left frontoparietal network, indicating that FC may partially contribute to compensate for disease-related structural damage and that it may gradually fail over time with the accrual of such damage (65). In adult RRMS, increased FC in bilateral frontoparietal networks was found in patients with preserved cognition, compared to healthy controls and patients with CI (66). On the other hand, in a large study including RR, SP, and primary progressive MS (PPMS), increased FC between thalamic and temporal regions (i.e., hippocampus, parahippocampal gyrus, superior temporal cortex) was found in patients with CI, compared to patients without CI, probably reflecting maladaptive mechanisms toward cognition (67). Moreover, default mode and frontoparietal networks showed increased FC with the rest of the brain in an MS population with CI including different phenotypes (RR, SP, and PP), suggesting that CI in MS may be due to abnormal communication of hub-rich networks (68). Decreased FC in the dorsal attention and default mode networks were also identified in adult RRMS patients with CI, probably reflecting a failure of compensatory mechanisms (66). In PPMS patients with CI, widespread seed-based functional network reorganization was found. In particular, there was decreased FC of the dorsal attention network with the insula and occipital cortex compared to PPMS patients without CI, whereas decreased FC of the executive control network with the insula and right frontoparietal network as well as between the dorsal attention network and the right frontoparietal network was observed compared to healthy controls (69).

#### SC–FC Coupling

In patients with clinically isolated syndrome (CIS), a stronger structural–functional coupling, reflected by the higher correlation coefficient between structural and functional networks (70), was able to predict worse global CI (70). This suggests that brain ability in reorganizing functional networks may diminish at later disease stages so that it can no longer compensate for MS-related structural damage (70).

#### Main Findings

- Decreased structural and functional network integration
- Increased structural and functional network segregation
- Altered FC in the default mode, dorsal attention, and frontoparietal networks.

### Attention

Approximately 10% of MS patients experience attention impairment (15), which could be evaluated by either Symbol Digit Modalities Test (SDMT) (71) or Paced Auditory Serial Addition Test (PASAT) (71). Basic attention tasks (i.e., repeating digits) are mostly unaffected in MS patients (3). Impairments are more common in sustained and divided attention, where patients are asked to attend several tasks simultaneously (3).

#### Structural Connectivity

Globally, in RRMS, lower PASAT correlated with measures of SC disruption such as reduced global and local efficiency and clustering coefficient (72). Meanwhile, reduced efficiency showed a close correlation with larger WM lesion volume (LV), underlying the role of lesions as a contributor to structural network disruption in RRMS (72). Another study showed that reduced global efficiency in SC may help explain decreased SDMT across different MS phenotypes (RR, SP, PP) (73).

Locally, in RR and SPMS, decreased nodal strength in the frontoparietal network, mainly driven by WM LV, correlated with worse PASAT, underlying the importance of the SC within such bilateral network (74).

#### Functional Connectivity

A reduction in the whole-brain static interhemispheric FC was able to explain well in RRMS worse attention, as measured by decreased SDMT and PASAT performances (75). In addition, better PASAT performance was associated with weaker whole-brain dynamic interhemispheric FC, suggesting that preserved attention in RRMS may be mediated by a smaller flexibility in such a type of connectivity (75).

Regionally, decreased FC in the dorsal attention and visual networks was shown in RRMS patients during a visual attention task (76). On the other hand, increased FC in the frontoparietal network, a hub-rich network, with the rest of the brain (both peripheral and nonhub regions) correlated with worse attention in an MS population including RR and progressive forms (68). Moreover, results of an interventional study showed in RRMS patients that, after 12-week computer-assisted rehabilitation of attention, FC within executive control, salience, and default mode networks increased and correlated with improved attention (77).

#### Main Findings

- Decreased structural network integration and segregation
- Altered SC and FC in the frontoparietal network.

### Information Processing Speed

IPS represents the amount of work performed within a time limit (e.g., number of items completed) (54) and is often assessed in MS by SDMT (71) or PASAT (71). IPS is the most commonly affected cognitive domain in all MS phenotypes (3), with a prevalence of 27 to 51% (15).

#### Structural Connectivity

At whole-brain level, it was shown in RRMS patients a reduced strength in rich-club and feeder (i.e., between hub and nonhub region) connections, reflecting widespread structural disconnection across the brain and a correlation of it with reduced IPS as measured by PASAT (78). In patients with CIS, increased structural clustering coefficient, reflecting the strengthening of short-distance connections preserving local information flow, correlated with worse IPS, as measured by a computerized speed cognitive test, a novel test for IPS (79). In RRMS, decreased efficiency (both global and local) and clustering coefficient across the brain correlated with lower PASAT (72). Moreover, in a heterogeneous MS population (RR, SP, PP), decreased global efficiency across the brain correlated with worse SDMT (73). Based on NODDI data, CIS patients showed that higher whole-brain modularity coefficient was associated with worse IPS as measured by SDMT (80). Of note, the standardized regression coefficient describing such relationship was greater when the modularity coefficient was obtained with NODDI data than with conventional DTI, indicating a better sensitivity of NODDI for MS (80).

Beyond whole-brain alterations, SC disruption in various structural networks showed cognitive relevance in MS. Module efficiency, which evaluates the communication efficiency both within and between structural networks, was found decreased in RRMS patients within visual network, between visual and deep GM networks, and between default mode and frontoparietal networks (45), and such reductions were correlated with lower PASAT (45). In another study, a close correlation between lower SDMT and reduced global efficiency in the default mode network was found in RRMS patients with and without CI, although the decrease in such network measure was more pronounced in the former group (81).

#### Functional Connectivity

Only one study assessed the relevance for IPS of the whole-brain FC, whose increase correlated with decreased IPS, as measured by SDMT, in a large and heterogeneous MS population (RR, SP, PP) (82).

The relevance for IPS of the frontoparietal and default mode networks was found not only for SC, as mentioned previously, but also for FC. Indeed, it was shown in an MS population with CI including different phenotypes (RR, SP, PP) that increased FC of these two networks with the rest of the brain correlated with worse IPS (68). During intrascanner SDMT, RR and SPMS patients with IPS impairment showed, compared to healthy subjects, an opposite direction of the effective connectivity in the frontoparietal networks (83). Specifically, the FC direction in such networks was from right dorsolateral prefrontal to right supplementary motor cortex and from right inferior parietal to left superior parietal cortex (83). In addition, in RRMS patients, a higher FC within the default mode network, specifically between medial prefrontal and frontal pole regions, appeared to facilitate performance stability during a computerized IPS test (84). The role of the default mode network in preserving IPS in RRMS patients was confirmed by the correlation between a larger increase in dynamic FC within such network from resting- to task-state and a better performance of intrascanner SDMT (85). Another study found that increased FC in the left frontoparietal network correlated with better IPS in both RR and SPMS patients (86). Moreover, an increased FC within the salience network, also involved in effective IPS, was found in RR and SPMS (86). Of note, in the same study, only in RRMS patients an FC increase within default mode network showed correlation with worse IPS (86).

In another study, worse IPS correlated with increased FC both within deep GM and between deep GM and cortex in advanced RRMS, and such a correlation further increased in SPMS (87).

After 8-week computer-aided cognitive rehabilitation, RRMS patients with CI showed IPS improvement in parallel to an increase in the default mode network FC at the level of the posterior cingulate and bilateral inferior parietal cortices (88).

#### Main Findings

- Decreased structural network integration.
- Increased structural network segregation.
- Altered SC and FC of the frontoparietal and default mode networks.

### Executive Control

Executive control refers to the cognitive ability needed for complex goal-directed behavior and adaptation to environmental changes or demands, including planning, anticipating outcomes, and appropriately directing resources (3). This cognitive domain can be evaluated by Delis–Kaplan Executive Function System Sorting test (89), Stroop word–color test (ST) (90), and Controlled Oral Word Association Test (91). A 15 to 28% of MS patients usually experience deficits in the executive control domain (15).

#### Structural Connectivity

Worse executive control in RRMS patients correlated with decreased structural nodal strength in the frontoparietal networks, deep GM structures and insula (74), and within sensorimotor, dorsal attention, left frontoparietal, and default mode networks (92). In another study on SPMS patients where structural networks were obtained using ICA, the component including disrupted supratentorial WM projection tracts and limbic association tracts showed correlation with worse executive control (93).

#### Functional Connectivity

At whole-brain level, better executive control correlated with both higher dynamics and stronger stationary FC in RRMS (94).

Alterations of regional FC also showed relevance for executive control in MS. Indeed, the presence of “extra effective” (i.e., absent in the FC pattern of healthy subjects) connections during ST resulted different across MS phenotypes (95). In particular, worse executive control correlated with lower FC from left posterior parietal to dorsal anterior cingulate in BMS and with higher FC from right to left insula in SPMS, whereas no correlation was found in RRMS. These findings may reflect the fact that these three MS phenotypes tend to use distinctive mechanisms during a demanding executive control task (95). Another study demonstrated that in RRMS patients with executive control impairment, improvement after computer-assisted cognitive rehabilitation was associated with increased FC between anterior cingulate and frontoparietal cortices of the corresponding network (96). In presence of worse executive control performance during ST, PPMS patients showed, compared to healthy subjects, reduced effective connectivity from left ventromedial prefrontal cortex and increased effective connectivity from left dorsolateral prefrontal cortex to regions of the right frontoparietal network (97), all these abnormalities having a probable maladaptive meaning.

#### Main Findings

Altered SC and FC of the frontoparietal networks.

### Working Memory

Working memory refers to the cognitive system that retains information in mind while performing complex tasks such as reasoning, comprehension, and learning (98). Working memory can be measured by various cognitive tests such as PASAT (9, 54), Letter–Number Sequencing, and Spatial Span subtests, and can be divided into two processing levels, namely, maintenance and manipulation (99). Impairment in working memory has been detected since the early MS stage (100) and across disease phenotypes (101). A 27 to 44% of MS patients showed a decline in working memory over time (3).

#### Structural Connectivity

An important role for working memory in MS was demonstrated by structural integrity of the frontoparietal network. Decreased FA along the left superior longitudinal fascicle, which is one of the major WM tracts in the left frontoparietal network, correlated with lower working memory in RRMS, because of the disruption of the connections to the prefrontal regions implicated in this cognitive domain (102). As an extension, RR and SPMS patients showing decreased global and local efficiency in the frontoparietal network also showed worse working memory (103). In addition, a study on ICA-based structural networks in SPMS suggested that microstructural damage, assessed by reduced FA, along the supratentorial WM projection and limbic association tracts may contribute to the working memory deficit (93).

#### Functional Connectivity

Patients with early MS (i.e., CIS and RRMS) showed increased whole-brain functional network modularity (i.e., diminished functional integration between separate functional modules), and this correlated with worse working memory (104). In RRMS patients, better working memory, as measured by PASAT, was associated with smaller flexibility (i.e., more stability) of the interhemispheric dynamic FC involving temporal regions, anterior cingulate gyrus, and parietal regions (75).

Two studies assessed the improvement in working memory performance after a targeted computerized cognitive training. In the first one, it was found in a small group of patients with juvenile MS a less decrease (i.e., a relative increase) in FC between the subcomponents of the default mode network, probably reflecting training-induced plasticity (105). In the second one, performed in adult RRMS patients, it was shown that increased FC between anterior cingulate cortex and right middle frontal gyrus correlated with better executive control, whereas between anterior cingulate cortex and right inferior parietal lobule correlated with better processing speed, with both mechanisms contributing to the improvement in working memory (96). After receiving high-frequency repetitive transcranial magnetic stimulation at the level of the right dorsolateral prefrontal cortex, a better working memory in RRMS patients was associated with increased FC between right dorsolateral prefrontal cortex and right caudate nucleus and bilateral paracingulate gyrus (106).

#### Main Findings

- Altered SC and FC of the frontoparietal networks.
- Altered FC of the default mode network.

### Long-Term Memory

It represents the ability to learn new information and recall them at a later time (3). Long-term memory is tested by Selective Reminding Test (SRT) (54), California Verbal Learning Test, and Brief Visuospatial Memory Test, Revised (BVMT-R) (4, 54). Impairment in this cognitive domain in MS has a prevalence of 40 to 65% (3).

#### Structural Connectivity

Hippocampus is the key region of memory in the human brain (107, 108). In CIS and RRMS patients, a decrease in SC, expressed by reduced FA and increased axial diffusivity, along perforant pathways, which connect entorhinal cortex to hippocampus, was found in those patients with memory impairment (109). In another study on RRMS patients assessing tractography-derived hippocampal memory network, worse memory performance was associated with reduction in various SC measures [network efficiency, right hippocampus nodal strength, streamline count, and communicability (i.e., efficiency of the information spread) across network] at the level of the medial temporal lobe, thalamus, insula, and occipital cortex (110).

#### Functional Connectivity

Altered hippocampal FC is also important for long-term memory deficit in MS. Indeed, RRMS patients with impairment in this cognitive domain showed, compared to healthy controls, decreased FC on the left hemisphere between hippocampus and various cortical regions (superior frontal gyrus, precuneus, posterior cingulate cortex lateral occipital gyrus, angular gyrus) (109) and, compared to memory-preserved MS patients, both increased FC between left hippocampus and right supramarginal gyrus and decreased FC between left hippocampus and right temporo-occipital fusiform/lingual gyrus (109). In another study on RR and SPMS, increased FC in the right posterior hippocampus turned out to be the best correlation of long-term memory impairment (111). Lower dynamic FC of the right hippocampus, in addition to higher static FC of this structure with the rest of the brain, was also able to explain an additional 13% of variance (24% in total) in worse long-term memory in RR and SPMS (112). Following a training with a modified Story Memory Technique in an MS population including different phenotypes (RR, SP, and PP), improvement in long-term memory correlated with increased FC between left hippocampus and cortical regions involved in visual memory and hubs of the default mode network (113). PPMS patients showed increased FC, assessed with seed-based approach, between the cerebellar lobule VIIb and right precentral gyrus, correlating with worse long-term memory measured by BVMT (114). Furthermore, this cerebellar FC reorganization was partially independent from cerebellar atrophy and was probably expression of a maladaptive functional rewiring (114).

#### SC–FC Coupling

In patients with CIS, stronger structural–functional coupling correlated with worse long-term memory, measured by the SRT-consistent long-term retrieval, suggesting the presence of an exhaustion of functional compensation to structural damage during the early MS stage (70).

#### Main Findings

- Altered SC and FC in the hippocampus

Table 1 summarizes the SC and FC substrates of the different cognitive domains in MS patients.

**Table 1:** Summary of the main findings from MRI studies in MS patients showing, for each impaired cognitive domain, structural connectivity (SC) damage and functional connectivity (FC) alterations at both global and local levels (when present).

	**Main findings in patients with CI (compared with HC and/or patients without CI)**	
**Impaired cognitive domain**	**SC damage**	**FC alterations**
Global cognition	Global ↓ Local efficiency and nodal strength (in 170 RR and 18 SPMS) (61) ↓ Network efficiency (in 133 RRMS) (63)	Global ↓ Mean network degree, global efficiency and hierarchy, ↑ path length, and assortativity (in 45 BMS, 121 RR, and 80 SPMS) (64) Local ↓ Frontoparietal network bilaterally (in 15 RRMS) (65) ↓ Dorsal attention and default mode networks (in 15 RRMS) (66) ↑ FC between thalamic subregions and temporal regions (in 136 RR, 42 SP, and 9 PPMS) (67) ↑ Default mode and frontoparietal networks with the rest of the brain (in 243 RR, 53 SP, and 36 PPMS) (5) ↓ FC: between dorsal attention network and the insula and occipital cortex, between executive control network and the insula and right frontoparietal network, between dorsal attention network and right frontoparietal network (in 13 PPMS) (69)
Attention	Global ↓ Efficiency and clustering coefficient (in 32 RRMS) (72)	Global ↑ Static and dynamic FC (in 25 RRMS) (75)
	Local ↓ Integrity of the frontoparietal network bilaterally (in 66 RR and 6 SPMS) (74)	Local ↓ FC in the dorsal attention network and ↑ FC in the ventral attention network during a visual attention task (in 23 RRMS) (76)
	↑ FA along connections from cingulate, frontal and occipital cortices (in 66 RR and 6 SPMS) (74)	↑ FC between frontoparietal network and the rest of the brain (both peripheral and nonhub regions) (in 243 RR, 53 SP, and 36 PPMS) (68)
Information processing speed	Global ↓ Rich-club organization (in 32 RRMS) (78) ↓ Efficiency and clustering coefficient (in 58 RR, 36 SP, and 28 PPMS) (73) ↑ Modularity coefficient (in 19 CIS) (80)	Global ↓ FC at whole-brain level and of the default mode and frontoparietal networks with the rest of the brain (in 83 RR, 31 SP, and 16 PPMS) (82)
	Local ↓ Module efficiency within visual network, between visual and deep GM networks and between default mode and frontoparietal networks (in 32 RRMS) (45) ↓ FA-weighted global efficiency of the default mode network, between visual and deep GM networks, and between default mode and frontoparietal networks (in 68 RRMS) (81)	Local ↓ Effective connectivity from right to left frontoparietal network during a processing speed task (in 16 RR, 3 SP, and 1 PPMS) (83) ↓ FC within default mode network between medial prefrontal and frontal pole regions facilitates performance stability (in 18 RRMS) (84) FC within default mode and salience networks and ↓ FC in the left frontoparietal network (in 40 RR and 25 SPMS) (86) ↑ FC within deep GM and between deep GM and cortex (in late 243 RR and 53 SPMS) (87)
Executive control	Global ↓ Nodal strength within sensorimotor, dorsal attention, left frontoparietal, and default mode networks (in 72 RRMS) (74)	Global ↓ Interplay between dynamic and stationary FC (in 46 RRMS) (94)
	Local ↓ Strength in the frontoparietal networks, deep GM and insula (in 33 RRMS) (92) ↓ FA in supratentorial projection and limbic association tracts (in 30 SPMS) (93)	Local ↓ FC from left posterior parietal to dorsal anterior cingulate (in 18 BMS) (95) ↑ FC from right to left insula (in 33 SPMS) (95) ↓ Effective connectivity from left ventromedial prefrontal cortex to right frontoparietal network, ↑ effective connectivity from left dorsolateral prefrontal cortex to right frontoparietal network (in 14 PPMS) (97)
Working memory		Global ↑ Whole-brain functional modularity (in 8 CIS and 8 RRMS) (104) ↑ Flexibility of interhemispheric dynamic FC between temporal regions, anterior cingulate gyrus, and parietal regions (in 25 RRMS) (75)
	Local ↓ FA along left superior longitudinal fascicle (in 23 RRMS) (102)	Local ↑ FC between default mode network components (in 5 juvenile MS after cognitive training) (105)
	↓ Efficiency in the frontoparietal network (in 91 RR and 11 SPMS) (103)	↑ FC between anterior cingulate cortex and right middle frontal gyrus, between anterior cingulate cortex and right inferior parietal lobule (in 17 RRMS after cognitive rehabilitation) (96)
Long-term memory	Local ↓ SC between entorhinal cortex and hippocampus (in 16 CIS and 15 RRMS) (109) ↓ SC measures (efficiency, strength, streamline count, and communicability) in the hippocampal network (in 71 RRMS) (110)	Local ↓ FC between left hippocampus and various cortical regions (superior frontal gyrus, precuneus, posterior cingulate cortex, lateral occipital gyrus, angular gyrus) ↑ FC between left hippocampus and right supramarginal gyrus ↓ FC between left hippocampus and right temporo-occipital fusiform/lingual gyrus (in 15 RRMS) (109) ↑ FC on the right posterior hippocampus (in 53 RR and 11 SPMS) (111) ↑ FC between the cerebellar lobule VIIb and right precentral gyrus (in 29 PPMS) (114) ↓ Dynamic FC of the right hippocampus,and ↑ static FC of the right hippocampus with the rest of the brain (in 30 RR and 8 SPMS) (112)

*BMS, benign MS; CI, cognitive impairment; CIS, clinically isolated syndrome; GM, gray matter; HC, healthy control; MS, multiple sclerosis; RR, relapsing–remitting; SP, secondary progressive; PP, primary progressive*.

Table 2 summarizes the findings of altered SC and FC of frontoparietal network across cognitive domains.

**Table 2:** Findings of SC damage and FC alterations of the frontoparietal network across cognitive domains in MS.

**Cognitive domain**	**Connectivity type**	**Connectivity findings**
Attention	SC	Decreased nodal strength
	FC	Increased FC
Information processing speed	SC	Decreased communication efficiency between frontoparietal and default mode networks
	FC	Increased FC
Executive control	SC	Decreased nodal strength
	FC	Extra effective connectivity to the right frontoparietal network
Working memory	SC	Decreased global and local efficiency
	FC	—
Long-term memory	SC	—
	FC	—

*SC, structural connectivity; FC, functional connectivity*.

### Cognitive Reserve

Cognitive reserve, which reflects the ability to cope with disease-related CI, is thought to explain in MS the incomplete relationship between brain disease and cognitive status (115, 116).

#### Structural Connectivity

Only recently, SC has been used to investigate cognitive reserve in MS. In a study, a moderate correlation between higher cognitive reserve index and more preserved graph measures of SC (nodal strength, global and local efficiency, cluster coefficient and transitivity) across the brain was observed in RR and SPMS patients with CI but not in those with preserved cognition, a finding that highlights the important protective role of cognitive reserve (117).

#### Functional Connectivity

A negative relationship between higher cognitive reserve index and lower FC within salience network and occipital regions was observed in RRMS (118). Moreover, RRMS patients with higher premorbid verbal intelligence, a proxy for cognitive reserve, exhibited preserved whole-brain FC despite progressive GM atrophy, stressing the role of preserved FC for a high level of cognitive reserve despite structural damage (119).

### Cognitive Phenotypes

The characterization of MS cognitive phenotypes may represent a step toward a better knowledge of the CI pathogenesis and personalized treatment (8). To date, there is no study assessing SC or FC in different MS cognitive phenotypes.

## Future Directions

New diffusion MRI techniques, such as DKI, DSI, HARDI, and NODDI, should be considered when assessing in MS the relevance toward CI of disconnection in brain regions with crossing fibers (21). Moreover, as MS lesions may affect the tractography-derived reconstruction of WM fibers, they need to be taken better into account. While traditional DTI-based fiber tracking may underestimate the effect of MS lesions on WM tracts, novel methods such as constrained spherical deconvolution–based fiber tracking (120, 121) and convex optimization modeling for microstructure informed tractography (122) were able to perform a more adequate WM fiber tracts reconstruction in the MS lesional brain (121, 122), thus providing a more reliable assessment of SC (122). Finally, data-driven methods for extracting structural networks, such as ICA and NMF, have rarely been used in the MS field. These methods provide a “soft” parcellation of the brain, where each voxel can contribute to build up multiple structural networks, thus being more sensitive to subtle pathology, whereas for “hard” parcellation, each voxel is uniquely assigned to a single structural network (31).

The field of FC appears fractionated because of the different analysis approaches, and this limits the replication and clinical translation of the various findings (35). In order to improve the clinical impact of FC, it is recommended for subsequent analysis and interpretation following a pipeline of “brain representation,” including both a spatial definition of brain units and a summary measure representing their different features (35).

To our knowledge, no study has ever assessed SC and FC in different cognitive MS phenotypes. Future studies in this field would help overcome the heterogeneity of CI in MS and better characterize cognitive groups with impairment in single or multiple domains (54).

Reorganization of both altered SC and FC, whether “compensatory” or “maladaptive,” is an important characteristic of MS (38, 123). However, evidence on cognition-related connectivity abnormalities in MS mostly derives from cross-sectional studies, and thus, it is difficult to claim whether such abnormalities may or may not be beneficial for cognitive performance of MS patients (123). Large prospective longitudinal studies of multimodal MRI are needed in MS in order to reveal relationships between worsening CI and changes over time in specific brain structures and functions (54).

“Fusion” methods (124), by considering the brain as a unified system, are able to simultaneously map alterations across different MRI modalities and include unsupervised multivariate methods such as independent component analysis (124), canonical correlation analysis, partial least-squares regression (125), and multilayer brain networks (126, 127). Such methods may be useful in shedding light on the joint mechanisms of altered SC and FC reorganization underlying CI in MS.

## Conclusions

In recent years, studies on SC and FC contributed to the understanding of MS-related CI. However, further studies are needed to make these abnormalities more easily interpretable in the research setting and above all useful in clinical practice, by taking into account the use of standardized pipelines and the possible bias introduced by MS lesions. Finally, longitudinal multimodal MRI studies may shed light on the changing associations between concurrent pathogenic mechanisms and MS-related CI.

## Author Contributions

All authors listed have made a substantial, direct and intellectual contribution to the work, and approved it for publication.

## Conflict of Interest

NS has received honoraria from Biogen-Idec, Bristol Myers Squibb, Celgene, Genzyme, Immunic, Merck Serono, Novartis, Roche and Teva for consulting services, speaking, and travel support. He serves on advisory boards for Merck, Novartis, Biogen-Idec, Roche, and Genzyme, Immunic and he has received research grant support from the Italian MS Society. The remaining authors declare that the research was conducted in the absence of any commercial or financial relationships that could be construed as a potential conflict of interest.
